# Stress Hormones Receptors in the Amygdala Mediate the Effects of Stress on the Consolidation, but Not the Retrieval, of a Non Aversive Spatial Task

**DOI:** 10.1371/journal.pone.0029988

**Published:** 2012-01-11

**Authors:** Amir Segev, Assaf Ramot, Irit Akirav

**Affiliations:** Department of Psychology, University of Haifa, Haifa, Israel; Alexander Flemming Biomedical Sciences Research Center, Greece

## Abstract

This study examined the effects of the arousal level of the rat and exposure to a behavioral stressor on acquisition, consolidation and retrieval of a non-aversive hippocampal-dependent learning paradigm, the object location task. Learning was tested under two arousal conditions: no previous habituation to the experimental context (high novelty stress/arousal level) or extensive prior habituation (reduced novelty stress/arousal level). Results indicated that in the habituated rats, exposure to an out-of-context stressor (i.e, elevated platform stress) impaired consolidation and retrieval, but not acquisition, of the task. Non-habituated animals under both stressed and control conditions did not show retention of the task. In habituated rats, RU-486 (10 ng/side), a glucocorticoid receptor (GR) antagonist, or propranolol (0.75 µg/side), a beta-adrenergic antagonist, injected into the basolateral amygdala (BLA), prevented the impairing effects of the stressor on consolidation, but not on retrieval. The CB1/CB2 receptor agonist WIN55,212-2 (WIN, 5 µg/side) microinjected into the BLA did not prevent the effects of stress on either consolidation or retrieval. Taken together the results suggest that: (i) GR and β-adrenergic receptors in the BLA mediate the impairing effects of stress on the consolidation, but not the retrieval, of a neutral, non-aversive hippocampal-dependent task, (ii) the impairing effects of stress on hippocampal consolidation and retrieval are mediated by different neural mechanisms (i.e., different neurotransmitters or different brain areas), and (iii) the effects of stress on memory depend on the interaction between several main factors such as the stage of memory processing under investigation, the animal's level of arousal and the nature of the task (neutral or aversive).

## Introduction

Exposure to stress, as well as the release of stress related hormones, plays a key role in regulating memory storage [1]–[4]. There is extensive evidence showing enhancing as well as impairing effects on learning and memory following acute stress or acute glucocorticoid treatment [1], [4]–[9]. Many reviews have discussed the role of the amygdala in modulating the storage of memory [10]–[11], including memory mediated by the hippocampus [2], [12]–[13]. It has been shown that the basolateral amygdala (BLA) is particularly important for mediating stress hormone and drug effects on memory consolidation in other brain regions [2], [14]–[16]. Recent studies show that antagonists of β-adrenergic receptors and glucocorticoid receptors (GRs) block the memory-enhancing effects of emotional arousal [17]–[19]. In a previous study we found that in a non-aversive object recognition task, animals perform differently under two conditions of arousal: extensive prior habituation to the experimental context (reduced novelty stress/arousal level) or no previous habituation (high novelty stress/arousal level). Exposure to an out-of-context stressor impaired consolidation in habituated animals but enhanced consolidation in the non-habituated ones [19]. Furthermore, the GR antagonist RU-486 microinjected into the BLA prevented the impairing (habituated rats) and enhancing (non-habituated rats) effects of the stressor. The β-adrenergic receptor antagonist propranolol microinjected into the BLA prevented the impaired performance of non-habituated control rats in the test. Compared with habituated rats, non-habituated rats show higher corticosterone levels and higher levels of anxiety [19]–[20].

Memory retrieval had also been found to be modulated by stress and stress hormones where the typical outcome is impaired performance. De Quervain et al. [21] showed that exposure to stress or corticosterone injection 30 minutes prior to testing impaired retrieval in the hippocampal-dependent Morris water maze task, and that blocking corticosterone synthesis prevents stress-induced impairment of retrieval. Furthermore, while activating GRs at the hippocampus prior to retrieval impaired retention of a spatial task, this was not evident when BLA GRs were activated [16]. However the water maze is a stressful learning experience, and therefore it might be problematic to use it to dissociate between out-of context stress and task associated arousal. In humans, [22] it was found that cortisone administered orally 1 hour prior to retrieval impaired verbal recall performance and that blockade of beta-adrenergic receptors prevents this glucocorticoid induced impairment [23].

In addition to the adrenal hormones, the cannabinoid system has been recently suggested as having an important part in regulating the stress response, particularly in the BLA [24]–[27]. Hence we also examined the effects of cannabinoid receptor activation in this region on stress-induced modulation of memory processes.

The hippocampal-dependent object location task [28]–[30] does not involve an explicit exogenous reinforcer. Because no rewarding or aversive stimulation is used during training, the learning occurs under conditions of relatively low stress or arousal. In a previous study, we examined arousal and stress effects on consolidation and reconsolidation of recognition memory [19]. The object recognition task is to a great extent dependent on the prefrontal cortex and the perirhinal cortex [31]–[32]. In the current study we are using the spatial version of the object recognition task that is a hippocampal-dependent memory task [28]–[29], [33] and thus may respond differently to stress and arousal than the visually-guided object recognition task.

There are several studies suggesting that the different memory stages are differently influenced by stress [34]. For example, stress exposure or GR activation may enhance the consolidation of hippocampal long-term memory, but impair memory retrieval and have no effect on memory acquisition [16], [35]–[36]. While other studies examined the effects of stress on memory processes in tasks with some degree of stress as the water maze [16], or in tasks relying on extra-hippocampal regions [19], or examined particular stages of memory [19], [35]–[36], we focused on three memory stages (acquisition, consolidation and retrieval) in a non-aversive hippocampal task, thus allowing to manipulate task related arousal and presenting an exogenous stressor without confounding these factors.

Hence we aimed: i) to examine whether exposure to stress would differentially affect the acquisition, consolidation and retrieval of the object location task. Learning was tested under two arousal conditions: no previous habituation to the experimental context (high novelty stress/arousal level) or extensive prior habituation (reduced novelty stress/arousal level), and ii) to examine whether antagonists of the stress hormones receptors microinjected into the BLA could prevent the effects of stress on performance.

## Results

**Figure 1:**
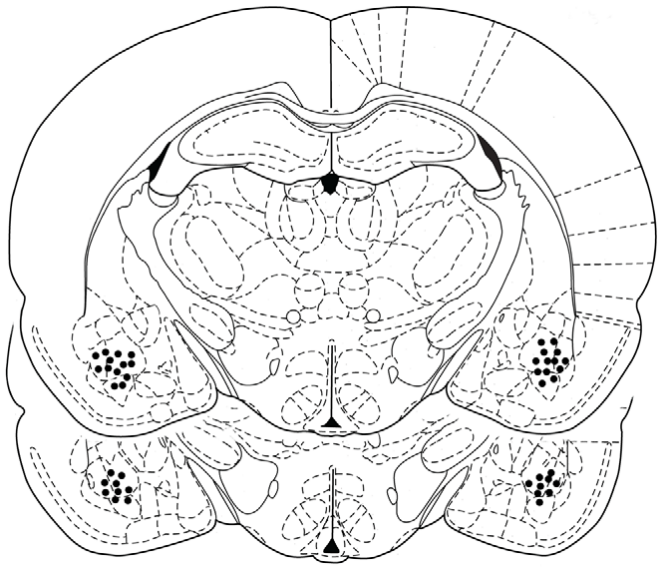
Representative schematic drawing of cannulae tips positions in the basolateral amygdala (BLA). Black circles show the representative locations of the cannulae tip at coronal views of the BLA (2.56 mm and 2.80 mm posterior to bregma).

**Figure 2:**
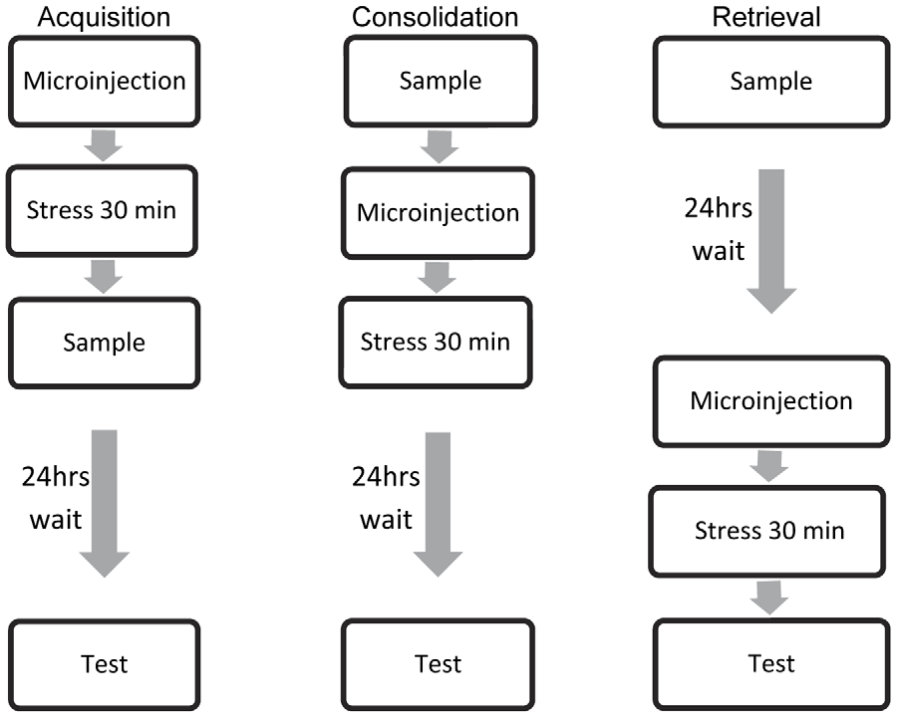
Experimental procedure for the individual experiments. In all the pharmacological experiments microinjection was preformed prior to exposure to stress.

Representative schematic drawing of cannulae tips positions in the BLA is shown in **Figure 1** and experimental procedure for the individual experiments is described in **Figure 2**.

**Figure 3:**
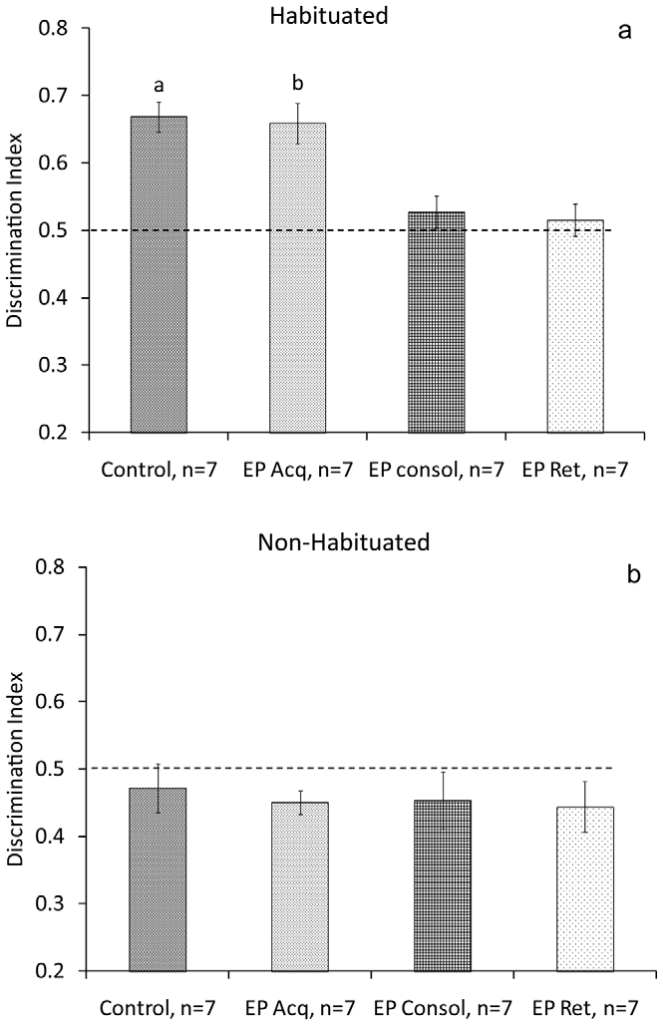
The effects of stress on acquisition, consolidation and retrieval in habituated and non-habituated rats. **a**. In habituated rats, the control group spent significantly more time exploring the new location compared with rats that were subjected to the stressor immediately after the sample phase (EP Consol) or immediately before the test phase (EP Ret). a, b: P<0.001: different from EP Consol and EP Ret groups. **b**. In non-habituated rats, all groups showed poor performance in the task.

In the first experiment we examined the effects of exposure to stress on the acquisition, consolidation and retrieval of the object location task in habituated and non-habituated rats. In habituated rats, ANOVA revealed a significant difference between the groups in discrimination index on day 2 (F_(3,24)_ = 10.644, P<0.001) (**Fig. 3a**). Post hoc comparisons revealed that the control group (n = 7) spent significantly more time exploring the new location compared with rats that were subjected to the stressor immediately after the sample phase (EP Consol, n = 7; P = 0.003) or immediately before the test phase (EP Ret, n = 7; P = 0.001). Furthermore, rats that were subjected to the stressor immediately before the sample phase (EP Acq, n = 7) spent significantly more time exploring the new location compared with the EP Consol (P = 0.006) and EP Ret (P = 0.003) groups. There was no significant difference between the groups in discrimination index during the sample phase (day 1) (F_(3,24)_<1, NS).

In non-habituated rats, ANOVA did not reveal a significant difference between the groups in discrimination index on day 2 (F_(3,24)_<1, NS) (**Fig. 3b**), or day 1 (F_(3,24)_<1, NS).

Two-way ANOVA preformed on total exploration time during the sample phase did not reveal a significant effect for habituation/no habituation (F_(1,24)_ = 1.72, NS), stress/no stress (F_(124)_<1, NS) or an interaction between these variables (F_(1,24)_ = 1.79,NS) (**Table 1**). In the test phase, two-way ANOVA preformed on total exploration time revealed a significant effect for habituation, (F_(1,48)_ = 16.56, p<0.001), but not for stress (F_(3,48)_<1, NS) or an interaction between these variables (F_(3,48)_ = 2.23, NS) (**Table 2**). Post hoc comparisons showed that habituated rats spent significantly more time exploring the objects (P<0.001) during the test. However, all groups showed exploration times higher than 20 s on the test phase and no differences were found between the different treatments in the habituated and non-habituated groups. This suggests that the exposure to the stressor had no discernible effects on locomotor activity or the normal tendency for exploration of objects.

**Table 1:** Total exploration times (sec.) during the sample phase.

	Time of Stress exposure	Habituated	Non-Habituated
*Control*	**-**	51.34 (4.15), n = 7	35.36 (5.41), n = 7
*Stress*	Acquisition	37.48 (7.26), n = 7	37.65 (6.77), n = 7

No significant differences in total exploration time were found between the different conditions during the sample phase. All groups showed total exploration times higher than 20 s. Data represent the means and SEM.

**Table 2:** Total exploration times (sec.) during the test phase.

	Time of Stress exposure	Habituated	Non-Habituated
*Control*	**-**	32.8 (5.65), n = 7	23 (2.88), n = 7
*Stress*	Acquisition	49.16 (7.54), n = 7	22.57 (3.12), n = 7
*Stress*	Consolidation	29.32 (2.13), n = 7	23.29 (2.90), n = 7
*Stress*	Retrieval	31.54 (5.67), n = 7	22.93 (2.30), n = 7

Habituated rats spent significantly more time exploring the objects during the test (P<0.01). All groups showed total exploration>20 s. Data represent the means and SEM.

In contrast to our initial hypothesis, stress did not facilitate the performance of non-habituated rats in this task (see discussion). However, in *habituated* rats, exposure to stress impaired consolidation and retrieval. Hence, we examined whether a GR antagonist microinjected into the BLA before stress exposure could block the effects of stress on the consolidation and retrieval of the task in habituated rats.

**Figure 4:**
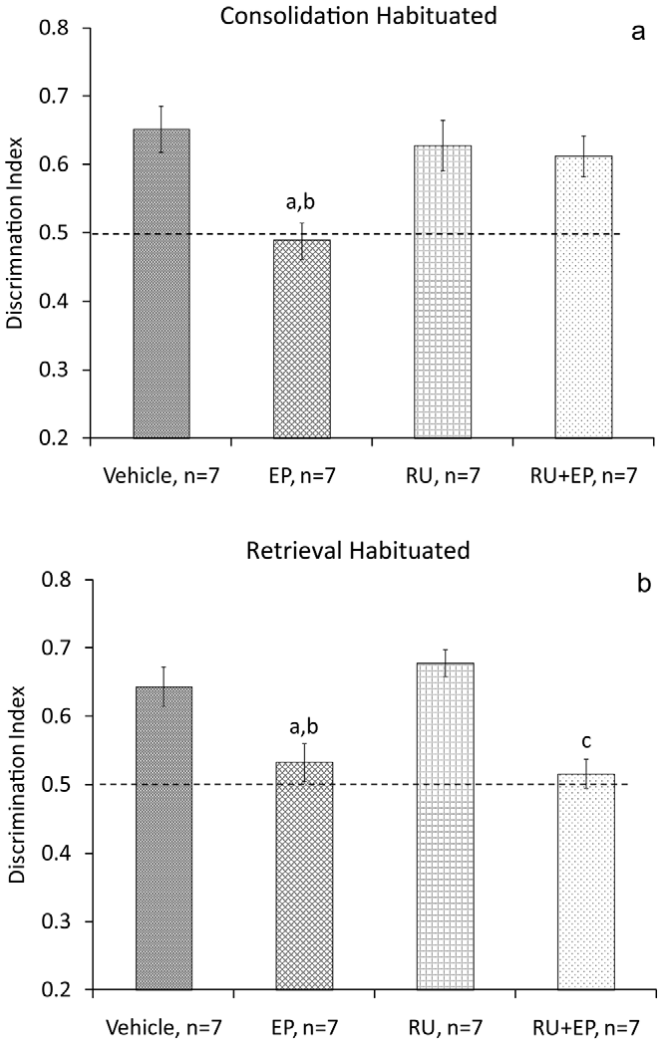
Intra-BLA RU-486 prevents the effects of stress on consolidation. **a**. RU-486 microinjected into the BLA prevents the effects of stress on consolidation of the object location task in habituated rats. a, P<0.001: different from Vehicle; b, P<0.05: different from RU and RU+EP groups. **b**. RU-486 microinjected into the BLA does not prevent the effects of stress on retrieval of the object location task in habituated rats. a, P<0.05: different from control, b, P<0.01 different from RU; c, P<0.01: different from control and RU groups.

Immediately after the sample phase, rats were microinjected with vehicle into the BLA and taken to their home cage (Vehicle, n = 7); microinjected with vehicle and subjected to the EP stress (EP, n = 7); microinjected with RU and subjected to the EP (RU+EP, n = 7); or microinjected with RU (RU, n = 7) without exposure to the stressor (**Fig. 4a**). ANOVA revealed a significant difference between the groups in discrimination index on day 2 (F_(3,24)_ = 5.145, P = 0.007). Post hoc comparisons revealed that the EP group spent significantly less time exploring the new location compared with all the other groups (Vehicle: P = 0.007; RU: P = 0.025; RU+EP: P = 0.05). There was no significant difference between the groups in discrimination index during the sample phase (F_(3,24)_<1, NS).

In another set of rats, before being tested on the 2^nd^ day, rats were microinjected with vehicle into the BLA (Vehicle, n = 7); microinjected with vehicle and subjected to the EP stress (EP, n = 7); microinjected with RU and subjected to the EP (RU+EP, n = 7); or microinjected with RU (RU, n = 7; **Fig. 4b**). ANOVA revealed a significant difference between the groups in discrimination index on day 2 (F_(3,24)_ = 10.639, P<0.001). Post hoc comparisons revealed that the RU+EP and EP groups spent significantly less time exploring the new location compared with the Vehicle (RU+EP:P = 0.006; EP: P = 0.019) and RU (RU+EP: P = 0.001; EP: P = 0.002) groups. There was no significant difference between the groups in discrimination index during the sample phase (F_(3,24)_<1, NS).

**Figure 5:**
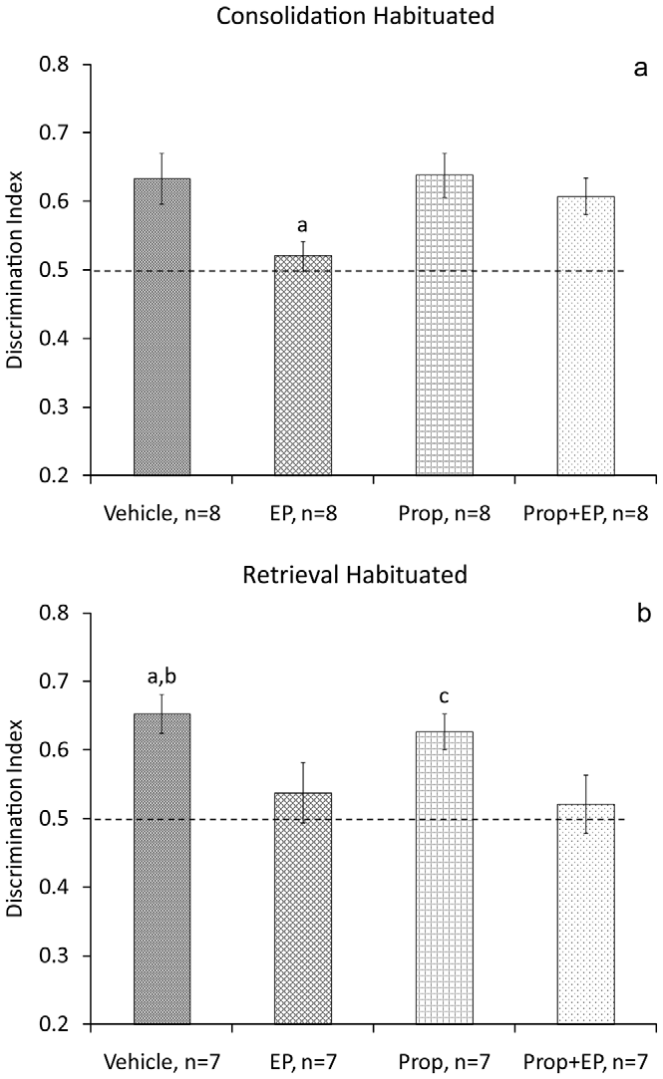
Intra-BLA propranolol prevents the effects of stress on consolidation. **a**. Propranolol microinjected into the BLA blocks the effects of stress on consolidation of the object location task in habituated rats. a, P<0.05: different from all groups. **b**. Propranolol microinjected into the BLA does not block the effects of stress on retrieval of the object location task in habituated rats. a, P<0.05: different from Prop+EP, b, c: P<0.01 different from EP.

Next we examined whether a beta-adrenergic antagonist microinjected into the BLA before stress exposure could block the effects of stress on the consolidation and retrieval of the task in habituated rats. Immediately after the sample phase, rats were microinjected with vehicle into the BLA and taken to their home cage (Vehicle, n = 8); microinjected with vehicle and subjected to the EP stress (EP, n = 8); microinjected with Prop and subjected to the EP (Prop+EP, n = 8); or microinjected with Prop (Prop, n = 8; **Fig. 5a**). ANOVA revealed a significant difference between the groups in discrimination index on day 2 (F_(3,28)_ = 3.351, P = 0.033). Post hoc comparisons revealed that the EP group spent significantly less time exploring the new location compared with all the other groups (Vehicle: P = 0.05; Prop: P = 0.044; Prop+EP: P = 0.05). There was no significant difference between the groups in discrimination index during the sample phase (F_(3,28)_<1, NS).

In another set of rats, before being tested on the 2^nd^ day, rats were microinjected with vehicle into the BLA (Vehicle, n = 7); microinjected with vehicle and subjected to the EP stress (EP, n = 7); microinjected with Prop and subjected to the EP (Prop+EP, n = 7); or microinjected with Prop (Prop, n = 7; **Fig. 5b**). ANOVA revealed a significant difference between the groups in discrimination index on day 2 (F_(3,24)_ = 3.219, P = 0.041). Post hoc comparisons revealed that the Vehicle group spent significantly more time exploring the new location compared with the EP (P = 0.002) and the Prop+EP (P = 0.025) groups. Also, the Prop group spent significantly more time exploring the new location compared with the EP group (P = 0.009). There was no significant difference between the groups in discrimination index during the sample phase (F_(3,24)_<1, NS).

We have recently found that cannabinoid receptor activation in the BLA using the CB1/2 receptor agonist WIN can prevent the stress-induced enhancement of inhibitory avoidance conditioning as well as the stress-induced disruption of extinction [24]. Intra-BLA WIN was also found to prevent trauma induced alterations in avoidance and acoustic startle response in a rat model of PTSD [37]. Hence, we aimed to examine here whether WIN microinjected into the BLA would block the effects of stress on performance in the object location task.

**Figure 6:**
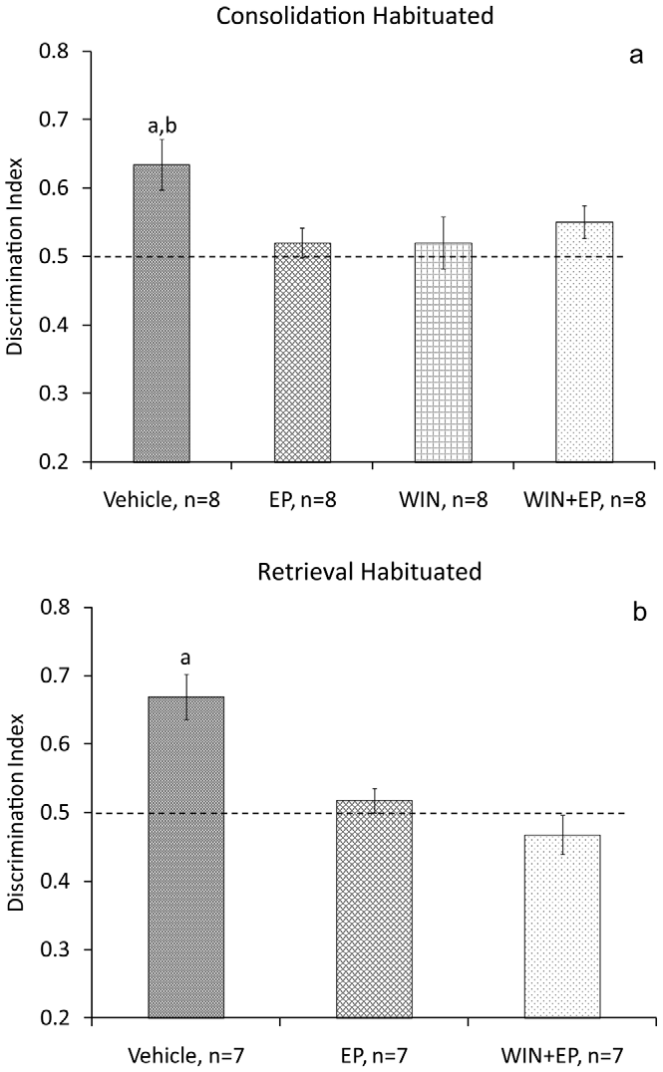
Intra-BLA WIN55,212-2 does not prevent the effects of stress on consolidation or retrieval. **a**. WIN55,212-2 microinjected into the BLA does not block the effects of stress on consolidation of the object location task in habituated rats. a, P<0.01: different from EP; P<0.05: different from WIN. **b**. WIN55,212-2 microinjected into the BLA does not block the effects of stress on retrieval of the object location task in habituated rats. a, P<0.01: different from all groups.

Immediately after the sample phase, rats were microinjected with vehicle into the BLA and taken to their home cage (Vehicle, n = 8); microinjected with vehicle and subjected to the EP stress (EP, n = 8); microinjected with WIN and subjected to the EP (WIN+EP, n = 8); or microinjected with WIN (WIN, n = 8; **Fig. 6a**). ANOVA revealed a significant difference between the groups in discrimination index on day 2 (F_(3,28)_ = 3.017, P = 0.046). Post hoc comparisons revealed that the Vehicle group spent significantly more time exploring the new location compared with the EP (P = 0.007) and WIN (P = 0.047) groups. As no significant difference was found between the vehicle and WIN+EP groups, we tested the difference between the discrimination index and the 0.5 chance level using a one-sample t-test. The vehicle group was significantly different from chance level (t_(7)_ = 3.625; P = 0.008), but not the WIN+EP group (t_(7)_ = 2.1, NS), suggesting that the WIN+EP group did not consolidate the task. There was no significant difference between the groups in discrimination index during the sample phase (F_(3,28)_<1, NS).

In another set of rats, before being tested on the 2^nd^ day, rats were microinjected with vehicle into the BLA (Vehicle, n = 7); microinjected with vehicle and subjected to the EP stress (EP, n = 7); or microinjected with WIN and subjected to the EP (WIN+EP, n = 7) (**Fig. 6b**). ANOVA revealed a significant difference between the groups in discrimination index on day 2 (F_(2,18_) = 14.669, P<0.001). Post hoc comparisons revealed that the Vehicle group spent significantly more time exploring the new location compared with the EP (P = 0.003) and WIN+EP (P<0.001) groups. There was no significant difference between the groups in discrimination index during the sample phase (F_(3,18)_<1, NS).

## Discussion

We found that exposure to a stressful experience impairs the consolidation and retrieval of a hippocampal-dependent non-aversive object location task in rats that were extensively habituated to the experimental context. Rats that had no previous habituation (high novelty stress or arousal level) did not show retention of the task indicated by poor discrimination on the test regardless of being stressed or not. GR and β-adrenergic receptors antagonists microinjected into the BLA before stress exposure prevented the impairing effects of stress on the consolidation, but not the retrieval, of the task. Finally, cannabinoid receptor activation in the BLA did not prevent the effects of stress on either the consolidation or retrieval of this task.

### The effects of stress exposure on the acquisition, consolidation and retrieval of the task

#### Consolidation

In a previous study [19], we found that exposure to stress impaired the consolidation of object recognition memory in habituated rats, corroborating with our current findings with the spatial object location task. However, in the previous study exposure to stress in non-habituated rats *enhanced* the consolidation of object recognition [19]. Both object recognition and object location tasks involve recognition memory which requires judgments concerning prior occurrence such as the relative familiarity of individual objects or locations, or the location of a previously encountered object, or when an object was previously encountered [28]. However, the object location task is hippocampal-dependent [28]–[30], [32] whereas the object recognition task is mostly dependent on the medial prefrontal and perirhinal cortex [31]–[32]. The poor performance of the non-habituated group in the object location task, regardless of exposure to a stressor, may stem from higher reactivity of the hippocampus to stress [38]–[40] compared with the prefrontal cortex-dependent object recognition task. Hence, while performance in both tasks may follow an inverted U-shape dose-dependency, where a moderate amount of stress is required for good performance, different curves exist for different tasks as these tasks rely on regions differentially susceptible to the effects stress.

When assessing the effects of stress or glucocorticoids levels of stress on consolidation, several studies show enhancing as well as impairing effects [19], [41]–[43]. This may heavily depend on the aversiveness of the learning experience. When the task is sufficiently aversive to activate the amygdala during memory consolidation, then an out-of-context stressor activates the same circuits as those activated by the stressful learning experience and memory consolidation is facilitated. However, during neutral or non-aversive tasks, the amygdala is not activated and an out-of-context stressor that activates the stress circuit (i.e., activates the amygdala and releases the stress hormones) may impair memory.

#### Acquisition

Exposure to stress had no effect on the acquisition of the object location task in habituated rats corroborating with previous studies showing that stress exposure or GR activation enhance the consolidation of hippocampal long-term memory, but impair memory retrieval and have no effect on memory acquisition [16], [21], [36], [43].

It should be noted that stress exposure at this time point might also affect consolidation processes. However, in our study stress exposure after the sample phase impaired consolidation with no effect when administered before the sample phase, strongly suggesting that stress exposure had no effect on acquisition.

It has been shown that stress levels of glucocorticoids prior to training impair acquisition of hippocampal-dependent tasks such as spatial memory in a water maze [44] and passive avoidance tasks [45]–[46]. In the object location task, the subject is presented and allowed to explore two identical objects. Following a delay, one of the objects is moved to a novel location. Hence, the role of the hippocampus relates to the spatial information necessary for task performance, for example identification of the spatial re-arrangement of familiar objects, rather than judgments of the familiarity of the objects themselves [29]. Accordingly, it is possible that stress had no effect on the acquisition of the object location task as this process is also dependent on other brain areas (e.g., prefrontal cortex and perirhinal cortex).

It is an intriguing question why a stronger stressor (EP) did not impair acquisition whereas a weaker stressor (i.e., new environment exposure in non-habituated rats) impaired it. Conrad [34] suggested that the learning function between glucocorticoids and hippocampal-dependent memory is modulated by 1) the aversive nature of the learning paradigm and 2) stage of memory processing (acquisition, consolidation, retrieval). Moreover, the direction of changes in memory performance also depend on whether the stress experienced is closely linked in time to and within the context of the information to be learned [47]. For example, in-context stressor (e.g. lower water temperature in the Morris water maze) will facilitate the acquisition of the spatial task [48], while an out-of-context stressor (e.g., footshock, elevated platform) experienced just before acquisition may impair of have no effect on performance. An interaction between these factors may explain why control non-habituated rats show impaired acquisition whereas exposure to the EP stressor had no effect on acquisition in habituated rats. When the stress is experienced in the context of a task that is highly aversive and the hormones and transmitters released in response to stress exert their actions on the same circuits as those activated by the situation, the result may be intact of facilitated performance. According to this rational, highly aroused non-habituated rats may show facilitated or intact performance in an aversive task. However, in non-habituated rats, the high arousal level does not correspond with the lack of aversiveness of the learning task and this results in impaired performance. This could explain why highly aroused rats do not acquire the neutral task.

#### Retrieval

There are other reports demonstrating impairment following stress exposure before retrieval [16], [21]. Thirty minutes after exposure to footshock stress, rats had impaired retrieval of spatial memory of a water-maze task they had acquired 24 h earlier [21]. It is possible that the memory retrieval impairment induced by the stressor is of temporary nature. There are reports that glucocorticoids impair the acute retrieval of contextual fear memory without affecting retrieval performance 48 h later [49]. Other reports suggest prolonged impairment of memory retrieval [50].

### Preventing the effects of stress on consolidation of the task

Intra-BLA microinjections of RU or propranolol after acquiring the task or before retrieving it did not induce memory impairments in the object location task. When microinjecting the antagonists into the BLA in habituated rats before stress exposure, RU and propranolol prevented the impairing effects of stress on consolidation, but not retrieval. It should be noted that although we used a small infusion volume (0.5 ml volume per side), the drugs may have spread to adjacent areas, such as the central nucleus of the amygdala.

Previously, it has been shown that the β-adrenoceptor antagonist propranolol (2.0 mg/kg) administered subcutaneously before retention testing did not affect retention performance alone, but blocked the memory retrieval impairment induced by concurrent intrahippocampal infusions of a GR agonist, RU 28362 [36]. In our study propranolol was administered locally into the BLA and did not block the effects of stress exposure on spatial retention. Three main differences between Roozendaal's study and ours that might explain this discrepancy are that (i) the effects of exposure to a stressor are likely less specific than those of a GR agonist microinjected into the hippocampus, (ii) the spatial task in the water maze is significantly more stressful than the spatial location task, and (iii) systemic administration of propranolol has probably affected other brain areas that are involved more directly in retrieval (e.g., the hippocampus) that are not directly affected by local injection into the BLA. Other studies have shown that systemic administration of propranolol blocks the memory retrieval impairment of spatial/contextual information induced by a concurrent injection of corticosterone [36] and that blockade of glucocorticoid production with the synthesis inhibitor metyrapone prevents stress-induced memory enhancement [51]. Taken together, the data suggest that the effects of stress on retrieval in the object location task are probably not mediated by the stress hormones receptors in the BLA.

In habituated rats, microinjecting RU or propranolol into the BLA prior to consolidation, but not prior to retrieval, prevented the impairing effects of stress on performance. If the drug was to affect the experience of stress (e.g., erase it) then rats microinjected with the antagonists and exposed to stress before retrieval should have shown intact performance of the task.

Cannabinoid receptor activation in the BLA did not prevent the effects of stress on consolidation or retrieval of the object location task. We have recently shown that cannabinoid receptor activation in the BLA blocked the effects of stress on conditioning and extinction of another hippocampal-dependent task (i.e., inhibitory avoidance) [24]. However, the memory phases that were tested were different as well as the nature of the task (i.e., highly aversive). Indeed, there are reports suggesting that cannabinoids modulate memory and stress-related behaviors under aversive conditions but not in non-aversive tasks [52]–[55].

### Summary

The effects of stress on memory depend on the interaction between several main factors: the memory stage under investigation, the animal's level of arousal and the nature of the task (neutral or aversive). We also show that the effects of stress on consolidation of a neutral hippocampal-dependent task are mediated by the stress hormones receptors in the BLA. The effects of stress on retrieval, on the other hand, are probably mediated by a different mechanism (e.g. different brain areas).

Most psychiatric disorders are associated with specific disturbances of consolidation and/or retrieval of memory, and stressful life events have been postulated to be important precursors of psychiatric illness including post-traumatic stress disorder, depression and addiction. Hence, understanding the characteristic neural and hormonal alterations that accompany the disturbances of consolidation and retrieval are of highly importance. Moreover, these results give preclinical support to the suggestion that stress hormones modulators may serve as possible therapeutics for the treatment of stress related disorders.

## Materials and Methods

### Ethics Statement

The experiments were approved by the University of Haifa Ethics and Animal Care Committee, and adequate measures were taken to minimize pain or discomfort (permit number: 116).

### Animals

Male Sprague-Dawley rats (Harlen, ∼60 day old, 250–300 g), group housed at 22±2°C under 12-h light/dark cycles. All rats were allowed free access to food and water.

### Drugs

The GR antagonist RU-38486 (RU; 10 ng/0.5 µl) and the β-adrenergic antagonist propranolol (Prop, 0.75 µg/0.5 µl) were obtained from Sigma (St Louis, MO). RU was first dissolved in 100% ethanol and subsequently diluted in saline to reach the appropriate concentration. The final concentration of ethanol was 2%. Controls were given the vehicle (2% ethanol) only. Prop was dissolved in physiological saline, which was also used as a control. The CB1/CB2 receptor agonist WIN55,212-2 (WIN, 5 µg/0.5 µl) (Tocris, USA) was dissolved in dimethylsulfoxide (DMSO) first, and diluted with saline (0.9% NaCl) and Tween-80 to the final volume. The concentration of DMSO was <1.5% in the final solution. Final Tween-80 concentration was 1%. Controls were given the vehicle only. Drug doses were based on previous work [19], [24].

### Cannulation and drug microinjection

Rats were anesthetized with 4.8 ml/kg Equithesin (2.12% w/v MgSO_4_ 10% ethanol, 39.1% v/v propylene glycol, 0.98% w/v sodium pentobarbital, and 4.2% w/v chloral hydrate), restrained in a stereotactic apparatus (Stoelting), and implanted bilaterally with a stainless steel guide cannula (23 gauge, thin walled) aimed at the BLA (anteroposterior, −3 mm; lateral, ±5 mm; ventral, −6.7 mm). The cannulae were set in place with acrylic dental cement and secured by two skull screws. A stylus was placed in the guide cannula to prevent clogging. Animals were allowed 1 week to recuperate before being subjected to experimental manipulations.

For microinjection, the stylus was removed from the guide cannula, and a 28 gauge injection cannula, extending 1.0 mm from the tip of the guide cannula, was inserted. The injection cannula was connected via polyethylene PE20 tubing to a Hamilton microsyringe driven by a microinfusion pump (PHD1000, Harvard Apparatus, USA). Microinjection was performed bilaterally in a 0.5 µl volume per side delivered over 1 min. The injection cannula was left in position for an additional 60 s before withdrawal to minimize dragging of the injected liquid along the injection tract. At all time points (i.e. prior to or following acquisition on day 1 and prior to retrieval on day 2) drugs were administered *before* exposure to the EP stressor.

### Elevated Platform Stress

Animals were placed on an elevated platform (EP; 12×12 cm) for 30 min in a brightly lit room [19], [24] The rats exhibit behavioral ‘freezing’, that is, immobility for up to 10 min, defecation, and urination.

### Habituation and no-habituation

Habituation for the experimental apparatus was performed by allowing rats to explore it for 5 min twice a day for 4 days before the experiment was performed (habituated). No object was placed inside the arena during habituation. The non-habituated groups were taken from their home cage with no habituation to the apparatus.

### Object location memory task

The objects were two plastic cups located in squared black open field (50×50×50 cm) under dim light and were glued firmly to the Plexiglas bottom, 10 cm from the walls. The open field and the objects were thoroughly cleaned between trials with odorous clean wipes.

In the sample phase (day 1), each rat was placed in the open-field arena and was exposed to the objects for two five-minute exploration sessions with a five minutes interval. These two sessions resulted in long-term memory. The test phase (day 2) was given 24 h after the sample trial. One object was moved to a new location and the time spent exploring the objects at the old and the new location was recorded for 5 min.

A CCD camera placed above the arena and connected to a video tape was used to track rat behavior during the exploration session. Recorded data was analyzed by two judges blind to experimental conditions and inter-rater reliability was assured.

Exploration was defined as when the subject sniffed at, whisked at, or looked at the object from no more than 2 cm away. A discrimination index calculated for each animal was expressed as *T*_N_/(*T*_N_+*T*_F_) (*T*_N_ = time spent exploring the object in the novel location; *T*_F_ = time spent exploring the object in the familiar location). Intact recognition memory in the test phase is reflected in a discrimination score higher than 0.5, which implies greater exploration of the object in the novel location.

Exposure to the stressor or microinjection of vehicle, RU, Prop or WIN, into the BLA took place at one of the following time points: immediately before the sample phase on day 1 (to test acquisition and or/consolidation), immediately after the sample phase on day 1 (to test consolidation), or immediately before the test phase on day 2 (to test retrieval). All figures show discrimination index in the test phase (day 2).

### Histology

At the completion of the behavioral experiments animals were euthanized by a lethal dose of sodium pentobarbital (300 mg/Kg) prior to decapitation and microinjected with 0.5 µL of India ink into the BLA. Brains were removed and brain slices (60 µm) were examined under a light microscope following Nissl staining to verify the cannula location. Approximately 10% of animals were excluded due to misplaced cannulae. Only data from animals with correct cannula placements were included in the analyses. **Figure 1** shows schematic drawing of BLA cannulae placements. Solid black circles indicate the locations in a subset of animals (not all animals are shown in light of the number of rats involved in the experiments).

### Statistics

Differences between the groups were determined using ANOVA and *t*-tests. All *post hoc* comparisons were made using Tukey.

## References

[1] de Kloet ER, Oitzl MS, Joëls M (1999). Stress and cognition: are corticosteroids good or bad guys?. Trends Neurosci.

[2] McGaugh JL (2002). Memory consolidation and the amygdala: a systems perspective.. Trends Neurosci.

[3] McIntyre CK, Marriott LK, Gold PE (2003). Cooperation between memory systems: Acetylcholine release in the amygdala correlates positively with performance on a hippocampus-dependent task.. Behav Neurosci.

[4] Roozendaal B (2002). Stress and memory: opposing effects of glucocorticoids on memory consolidation and memory retrieval.. Neurobiol Learn Mem.

[5] Akirav I, Sandi C, Richter-Levin G (2001). Differential activation of hippocampus and amygdala following spatial learning under stress.. Eur J Neurosci.

[6] Diamond DM, Fleshner M, Ingersoll N, Rose G (1996). Psychological stress impairs spatial working memory: Relevance to electrophysiological studies of hippocampal function.. Behav Neurosci.

[7] Lupien SJ, McEwen BS (1997). The acute effects of corticosteroids on cognition: integration of animal and human model studies.. Brain Res Rev.

[8] Sandi C, Rose SPR (1997). Training-dependent biphasic effects of corticosterone in memory formation for a passive avoidance task in chicks.. Psychopharmacology.

[9] Trnečková L, Hynie S, Šída P, Hliňák Z, Krejčí I (2005). Effects of Stress and of Amphetamine on Passive Avoidance Conditioning in Rats.. Gen Physiol Biophys.

[10] Cahill L, McGaugh JL (1996). Modulation of memory storage.. Curr Opin in Neurobiol.

[11] McGaugh JL, Roozendaal B (2002). Role of adrenal stress hormones in forming lasting memories in the brain.. Curr Opin Neurobiol.

[12] Roozendaal B, Lengvilas R, McGaugh JL, Civelli O, Reinscheid RK (2007). Orphanin FQ/nociceptin interacts with the basolateral amygdala noradrenergic system in memory consolidation.. Learn Mem.

[13] Richter-Levin G, Akirav I (2000). Amygdala-hippocampus dynamic interaction in relation to memory.. Mol Neurobiol.

[14] Packard MG, Cahill L, McGaugh JL (1994). Amygdala modulation of hippocampal-dependent and caudate nucleus-dependent memory processes.. Proc Natl Acad Sci U S A.

[15] Akirav I, Richter-Levin G (2002). Mechanisms of amygdala modulation of hippocampal plasticity.. J Neurosci.

[16] Roozendaal B, Griffith QK, Buranday J, de Quervain DJ, McGaugh JL (2003). The hippocampus mediates glucocorticoid-induced impairment of spatial memory retrieval: Dependence on the basolateral amygdale.. Proc Natl Acad Sci U S A.

[17] Cahill L, Prins B, Weber M, McGaugh JL (1994). β-Adrenergic activation and memory for emotional events.. Nature.

[18] van Stegeren AH, Everaerd W, Cahill L, McGaugh JL, Gooren LJG (1998). Memory for emotional events: differential effects of centrally versus peripherally acting β-blocking agents.. Psychopharmacology.

[19] Maroun M, Akirav I (2008). Arousal and stress effects on consolidation and reconsolidation of recognition memory.. Neuropsychopharmacology.

[20] Okuda S, Roozendaal B, McGaugh JL (2004). Glucocorticoid effects on object recognition memory require training-associated emotional arousal.. Proc Natl Acad Sci U S A.

[21] De Quervain DJ, Roozendaal B, McGaugh JL (1998). Stress and glucocorticoids impair retrieval of long-term spatial memory.. Nature.

[22] de Quervain DJ, Roozendaal B, Nitsch RM, McGaugh JL, Hock C (2000). Acute cortisone administration impairs retrieval of long-term declarative memory in humans.. Nat Neurosci.

[23] de Quervain DJ, Aerni A, Roozendaal B (2007). Preventive effect of beta-adrenoceptor blockade on glucocorticoid-induced memory retrieval deficits.. Am J Psychiatry.

[24] Ganon-Elazar E, Akirav I (2009). Cannabinoid receptor activation in the basolateral amygdala blocks the effects of stress on the conditioning and extinction of inhibitory avoidance.. J Neurosci.

[25] Hill MN, McLaughlin RJ, Morrish AC, Viau V, Floresco SB (2009). Suppression of amygdalar endocannabinoid signaling by stress contributes to activation of the hypothalamic-pituitary-adrenal axis.. Neuropsychopharmacology.

[26] Phan KL, Angstadt M, Golden J, Onyewuenyi I, Popovska A (2008). Cannabinoid modulation of amygdala reactivity to social signals of threat in humans.. J Neurosci.

[27] Akirav I (2011). The role of cannabinoids in modulating emotional and non-emotional memory processes in the hippocampus.. Front Behav Neurosci.

[28] Ennaceur A, Aggleton JP (1994). Spontaneous recognition of object configurations in rats: effects of fornix lesions.. Exp Brain Res.

[29] Mumby DG, Glenn MJ, Nesbitt C, Kyriazis DA (2002). Dissociation in retrograde memory for object discriminations and object recognition in rats with perirhinal cortex damage.. Behav Brain Res.

[30] Goodman T, Trouche S, Massou I, Verret L, Zerwas M (2010). Young hippocampal neurons are critical for recent and remote spatial memory in adult mice.. Neuroscience.

[31] Akirav I, Maroun M (2006). Ventromedial Prefrontal Cortex Is Obligatory for Consolidation and Reconsolidation of Object Recognition Memory.. Cereb Cortex.

[32] Warburton EC, Brown MW (2010). Findings from animals concerning when interactions between perirhinal cortex, hippocampus and medial prefrontal cortex are necessary for recognition memory.. Neuropsychologia.

[33] Ennaceur A, Neave N, Aggleton JP (1997). Spontaneous object recognition and object location memory in rats: the effects of lesions in the cingulate cortices, the medial prefrontal cortex, the cingulum bundle and the fornix.. Exp Brain Res.

[34] Conrad CD (2005). The relationship between acute glucocorticoid levels and hippocampal function depends upon task aversiveness and memory processing stage.. Nonlinearity Biol Toxicol Med.

[35] Roozendaal B, McGaugh JL (1997). Glucocorticoid receptor agonist and antagonist administration into the basolateral but not central amygdala modulates memory storage.. Neurobiol Learn Mem.

[36] Roozendaal B, De Quervain DJ, Schelling G, McGaugh JL (2004). A systemically administered beta-adrenoceptor antagonist blocks corticosterone-induced impairment of contextual memory retrieval in rats.. Neurobiol Learn Mem.

[37] Ganon-Elazar E, Akirav I (2011). Cannabinoids prevent the development of behavioral and endocrine alterations in a rat model of intense stress.. Neuropsychopharmacology.

[38] Garcia R, Musleh W, Tocco G, Thompson RF, Baudry M (1997). Time-dependent blockade of STP and LTP in hippocampal slices following acute stress in mice.. Neurosci Lett.

[39] Maroun M, Richter-Levin G (2003). Exposure to acute stress blocks the induction of long-term potentiation of the amygdala-prefrontal cortex pathway in vivo.. J Neurosci.

[40] Lupien SJ, Lepage M (2001). Stress, memory, and the hippocampus: can't live with it, can't live without it.. Behav Brain Res.

[41] Roozendaal B, McGaugh JL (1996). The memory-modulatory effects of glucocorticoids depend on an intact stria terminalis.. Brain Research.

[42] Roozendaal B, Okuda S, de Quervain DJ, McGaugh JL (2006). Glucocorticoids interact with emotion-induced noradrenergic activation in influencing different memory functions.. Neuroscience.

[43] Roozendaal B (1999). Curt P. Richter award. Glucocorticoids and the regulation of memory consolidation.. Psychoneuroendocrinology.

[44] Oitzl MS, de Kloet ER (1992). Selective corticosteroid antagonists modulate specific aspects of spatial orientation learning.. Behav Neurosci.

[45] Bohus B (1970). Central nervous structures and the effect of ACTH and corticosteroids on avoidance behavior: a study with intracerebral implantation of corticosteroids in the rat. Prog.. Brain Res.

[46] Kovács GL, Telegdy G, Lissák K (1977). Dose-dependent action of corticosteroids on brain serotonin content and passive avoidance behavior.. Horm Behav.

[47] Joëls M, Zhenwei P, Wiegert O, Oitzl MS, Krugers HJ (2006). Learning under stress: how does it work?. Trends Cogn Sci.

[48] Akirav I, Sandi C, Richter-Levin G (2001). Differential activation of hippocampus and amygdala following spatial learning under stress.. Eur J Neurosci.

[49] Cai WH, Blundell J, Han J, Greene RW, Powell CM (2006). Postreactivation glucocorticoids impair recall of established fear memory.. J Neurosci.

[50] Tollenaar MS, Elzinga BM, Spinhoven P, Everaerd W (2009). Immediate and prolonged effects of cortisol, but not propranolol, on memory retrieval in healthy young men.. Neurobiol Learn Mem.

[51] Liu L, Tsuji M, Takeda H, Takada K, Matsumiya T (1999). Adrenocortical suppression blocks the enhancement of memory storage produced by exposure to psychological stress in rats.. Brain Res.

[52] Hölter SM, Kallnik M, Wursta W, Marsicano G, Lutz B (2005). Cannabinoid CB1 receptor is dispensable for memory extinction in an appetitively-motivated learning task.. Eur J Pharmacol.

[53] de Oliveira Alvares L, Genro BP, Vaz Breda R, Pedroso MF, Da Costa JC (2006). AM251, a selective antagonist of the CB1 receptor, inhibits the induction of long-term potentiation and induces retrograde amnesia in rats.. Brain Res.

[54] Pamplona FA, Takahashi RN (2006). WIN 55212-2 impairs contextual fear conditioning through the activation of CB1 cannabinoid receptors.. Neurosci Lett.

[55] Niyuhire F, Varvel SA, Thorpe AJ, Stokes RJ, Wiley JL (2007). The disruptive effects of the CB1 receptor antagonist rimonabant on extinction learning in mice are task-specific.. Psychopharmacology.

